# Folate Deficiency Increased Lipid Accumulation and Leptin Production of Adipocytes

**DOI:** 10.3389/fnut.2022.852451

**Published:** 2022-04-25

**Authors:** Chun-Wai Chan, Pei-Hsuan Chan, Bi-Fong Lin

**Affiliations:** Department of Biochemical Science and Technology, College of Life Science, National Taiwan University, Taipei, Taiwan

**Keywords:** folate, leptin, adipocytes, lipogenesis, high-fat diet (HFD), high-fructose diet

## Abstract

Imbalanced dietary habits are closely associated with poor micronutrients status and the development of obesity. Previous studies have shown that serum folate level is decreased in obese individuals. However, whether folate deficiency could result in adiposity is still unclear. The aim of this study was to investigate the effects of dietary folate on lipid accumulation and leptin production using both *in vivo* and *in vitro* studies. Male C57BL/6 mice were fed with a diet with (f1) or without (f0) folate in a high-fat (HF) diet containing high-sucrose (HFS-f1, HFS-f0) for 4.5–5 months in Experiment 1, or an HF diet (HF-f1, HF-f0) for 12 months in Experiment 2, or an HF diet containing high-fructose (HFF-f1, HFF-f0) for 12 months in Experiment 3, compared with the normal-fat (NF-f1, NF-f0) diet, respectively. The serum levels of folate and leptin, white adipose tissue (WAT), size of adipocytes, hepatic contents of triglyceride (TG), and cholesterol were measured. *In vitro* study, TG contents, proinflammatory cytokines, leptin, and expressions of hypoxia-inducible factor (HIF)-1α and lipogenesis-related genes of 3T3-L1 adipocytes cultured with (f_1_) or without (f_0_) folate were assayed. The results showed that folate deficiency together with a high-fat diet (HFS-f0, HF-f0, HFF-f0) had higher WAT mass, adipocyte size, serum leptin level, and hepatic TG compared to those of the folate-sufficient groups (HFS-f1, HF-f1, and HFF-f1). Folate deficiency with a high-fat high -sucrose or -fructose diet (HFS-f0, HFF-f0) significantly increased the body weight of the mice. Increased intracellular TG, leptin, monocyte chemotactic protein (MCP)-1 and interleukin (IL)-6 levels, and the expression of *Hif1*α and lipogenesis-related genes *Cebp*α, *Cebp*β, *Acc1, Fasn*, and *Fabp4* were also detected in folate-deficient 3T3-L1 adipocytes. Our results suggested that folate deficiency increased lipid accumulation and leptin production of adipocytes, and thus, inadequate folate status might be one of the risk factors for adiposity.

## Introduction

Obesity is an epidemic chronic illness that occurs because of genetic factors, a sedentary lifestyle, a nutritional transition to manufactured food, and a high-calorie diet, or psychological effects (1). The prevalence of obesity has increased rapidly both in children and in adults up to > 30% in many countries over the past 30–50 years (2, 3). Obesity might frequently result in premature disability and death by increasing the risk of metabolic diseases such as type 2 diabetes and fatty liver disease, cardiovascular diseases (hypertension and stroke), depression, and a variety of cancers (breast, prostate, liver, and colon) (3). Adipose tissue mass is determined by an enlargement in adipocyte size (hypertrophy) and/or an increase in adipocyte numbers (hyperplasia). Adipocyte within white adipose tissue (WAT) plays a crucial role in energy metabolism for the time of energy storage and release. In response to long-term excessive caloric intake conditions, adipocytes chiefly expanded the cell size to fulfill the need for increased lipid storage. However, adipocytes cannot expand indefinitely, due to cell and tissue expansion limitations. Reaching an expansion threshold may cause adipose tissue hypoxic stress, subsequent activated hypoxia-inducible factor (HIF)-1α, a transcription factor in response to the hypoxic cellular stress due to an inappropriate supply of oxygen within the expanded adipocytes. Stressed adipocytes also contribute to inflammation, dysfunction, and ectopic and visceral lipid deposition (4, 5).

White adipose tissue also acts as an important endocrine and immunologic organ (6). Leptin, first known as a satiety hormone, is predominantly secreted by the adipocytes and thus found to be positively correlated with body fat percentage (7). In addition to the hormone characteristic, leptin is also classified as a cytokine-like peptide because it has high structural and functional similarities with interleukin (IL)-6 (8). Hyperleptinemia may be caused by leptin resistance, which further leads to chronic low-grade inflammation (9, 10). Therefore, increased leptin secretion by adipocytes, or adipose tissue, might lead to chronic inflammatory diseases.

Both the high-calorie diet-induced obesity murine model and mouse 3T3-L1 cell line have been commonly used to investigate the mechanisms of adipogenesis and lipogenesis (11). The progression of preadipocyte differentiation, such as 3T3-L1 fibroblasts, can be presented as the serial transcriptional factors. First, CCAAT/enhancer binding proteins (C/EBP) β and δ are expressed in preadipocytes during the early phase of differentiation. Activated C/EBPβ and δ mediate the expression of peroxisome proliferator-activated receptor-gamma (PPARγ) and C/EBPα in an intermediate phase. PPARγ and C/EBPα act alone or interact with each other to induce the transcription of various lipogenic genes during the late phase, such as acetyl-CoA carboxylase 1 (ACC1), fatty acid synthase (FASN), and fatty acid-binding protein 4 (FABP4) (12). The expression of these genes is involved in maintaining the adipocyte phenotype and fat storage. High glucose provides a carbon source to form fatty acid in adipocytes, namely, *de novo* lipogenesis. ACC1 catalyzes the first step of fatty acid synthesis to form malonyl-CoA and then into palmitate after each two-carbon addition by FASN (13). FABP4, also called adipocyte protein 2, is a lipid chaperone protein and is highly expressed in mature adipocytes to facilitate lipid droplets deposit (14). It is well-known that excessive energy nutrients promote lipid formation. However, whether the nutritional status of micronutrients could affect adipose tissue lipogenesis is less investigated. Since micronutrient deficiencies have been found in obese individuals in different populations, further studies are needed to clarify the roles of micronutrient deficiencies with respect to obesity (15).

Modern lifestyle tends to have imbalanced dietary habits, which include more palatable food that is high in fat, sugar, or fructose syrup, while less vegetable and fruit intake (16), and thus accompanies obesity and poor folate nutritional status (17). Folate is an essential nutrient for purine and thymidylate biosynthesis, cell division, and maintaining physiological homeostasis. The classical symptom of folate deficiency is megaloblastic anemia and low folate status is a risk factor for cancer (18). In addition, the influence of folate status on adipogenesis and lipogenesis has been less studied. Theoretically, folate deficiency inhibits cell division and may affect adipose tissue mass. Nevertheless, lower serum folate levels were noted in those childbearing-aged women with higher body mass index (BMI) in the US National Health and Nutrition Examination Survey (NHANES) (19). The association between adiposity and lower serum folate level has been reported in a study with postmenopausal women (20). Lower serum folate concentration and folate intake in healthy subjects with overweight and obesity were also observed in a recent study (21). However, these associations that were only demonstrated by epidemiological data could not reflect the cause and effect. Therefore, the effect of folate deficiency on adipose tissue formation is worthy of investigation.

Adiposity can be induced by not only a high-fat diet (HFD) but also excessive amounts of simple sugar consumption. Sugar or high fructose corn syrup-sweetened beverages have become increasingly popular over the past decades. Excessive consumptions of added sugars are a risk factor for developing obesity and intra-abdominal or visceral fat formation in animals and humans (22). Fructose-supplemented HFD-fed mice were noted to develop more pronounced obesity as compared to that of glucose-supplemented HFD (23), suggesting enhanced effects of fructose on lipogenesis. To investigate the effect of folate deficiency on adiposity, we used diet-induced obese mice in this study. First, we fed mice with HFD with high-sucrose for short-term folate deficiency to determine the adipose tissue formation under the combination of a high-fat and high-sucrose diet. Then, mice fed HFD with long-term folate deficiency were examined to confirm the effects of folate deficiency on adiposity. Finally, mice were fed with HFD containing high-fructose to investigate whether synergistic effects exist between high-fructose and folate deficiency. Our data demonstrated that folate deficiency increased fat mass, adipocyte size, higher serum leptin level, and hepatic triglyceride (TG) accumulation. The effects of folate deficiency on lipogenesis were further confirmed by 3T3-L1 adipocytes with higher expression of lipogenic genes.

## Materials and Methods

All the cell culture and molecular biology grade chemicals were purchased from Sigma Aldrich (St. Louis, MO, USA), otherwise specified.

### Animals and Diets

**Figure 1:**
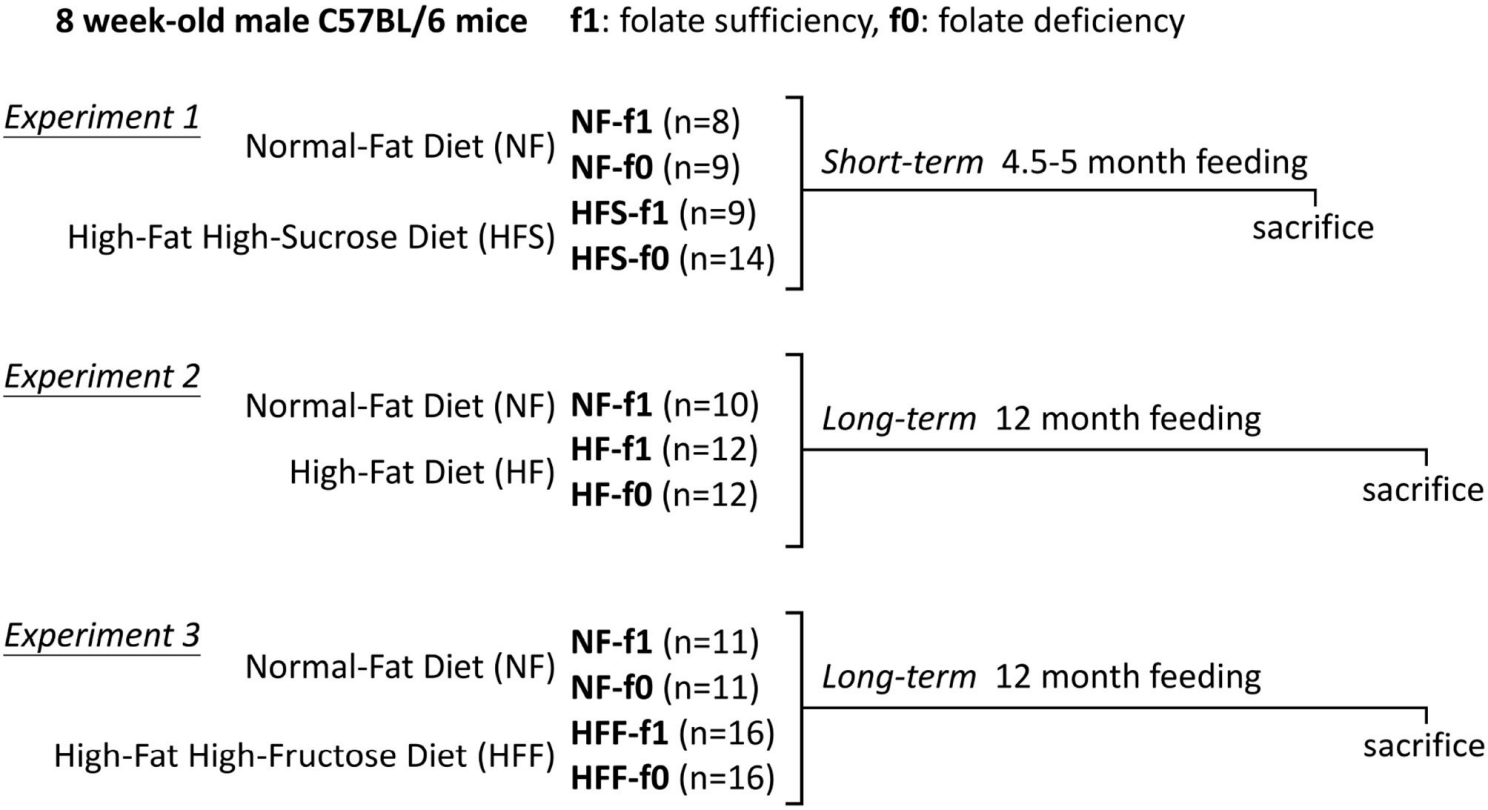
The experimental design for animal study.

The 6-week-old male C57BL/6 mice were purchased from National Laboratory Animal Center (Taipei, Taiwan, ROC) and single housed in cages with *ad libitum* access to AIN-93 diet and water, maintained on a 12-h light/12-h dark cycle under controlled environment at 23 ± 2°C and 55 ± 5% relative humidity. After 2 weeks of acclimatization, mice were randomly assigned into different groups. The experimental design for the animal study is shown in Figure 1. For Experiment 1, mice were fed either a normal-fat (NF; 16% Kcal fat) diet with (NF-f1) or without folate (NF-f0) or a high-fat high-sucrose (HFS; 54% Kcal fat) diet with (HFS-f1) or without folate (HFS-f0) for 4.5–5 months for a short-term study. For long-term experiments, mice were fed either an NF or high-fat (HF; 45% Kcal fat) diet with (NF-f1, HF-f1) or without folate (NF-f0, HF-f0) for 12 months in Experiment 2. Unfortunately, the NF-f0 group mice showed decreased feed intake and body weight and did not survive for 12 months. For Experiment 3, mice were fed either an NF or high-fat high-fructose (HFF; 45% Kcal fat) diet with (NF-f1, HFF-f1) or without folate (NF-f0, HFF-f0) for 12 months. To avoid the unexpected data loss of NF-f0 mice, folic acid (0.2 mg/kg diet, one-tenth of f1) was added back to the NF-f0 diet when the decrease in feed intake and body weight of NF-f0 group mice was observed after 1-month feeding. The composition of each purified diet is shown in Supplementary Table 1. The body weight and feed intake of each mouse were recorded at least once a week. Feed efficiency percentage (%) was calculated as the ratio of body weight gain (gram) to feed intake (gram) to compare the weight gains of mice fed with different compositions of diet. The animal protocols were reviewed and approved by the Institutional Animal Care and Use Committee (IACUC) of the National Taiwan University (approval no: NTU105-EL-17 and NTU107-EL-235).

### Histological Examination

Epididymal white adipose tissue (eWAT), subcutaneous WAT (sWAT), perirenal (rWAT), mesenteric WAT (mWAT), and liver tissues were collected from the mice at sacrifice. The eWAT and liver were rinsed with ice-cold phosphate-buffered saline (PBS), fixed with a 10% formalin solution. Paraffin-embedded sections were stained with hematoxylin and eosin (H&E). Images were captured on an IM-3 LED light microscope (Optika, Bergamo, Italy) equipped with the 8.3-megapixel digital camera (Optika) using the ProView software (Optika). Then, the adipocyte size was quantified using ImageJ software.

### Cell Culture

Murine 3T3-L1 cells were obtained from Bioresource Collection and Research Center (BCRC, Hsinchu, Taiwan, ROC). Cells were seeded on the 6-well-plate at 1 × 10^5^ cells/well and cultured in Dulbecco's modified eagle medium (DMEM; Himedia, Mumbai, India) supplemented with 10% heat-inactivated bovine serum (Gibco, ThermoFisher, Grand Island, NY, USA). When reaching full confluence, cells were cultured with the differentiation medium composed of 0.2 μM dexamethasone, 0.5 mM 3-isobutyl-1-methylxanthine, and 10 μg/ml insulin in 10% heat-inactivated fetal bovine serum (Gibco, ThermoFisher) DMEM. This differentiation medium was removed after 4 days and replaced with folic acid-free DMEM (Himedia) plus 10 μg/ml insulin either containing 0 μM (f_0_), 9.1 μM (f_1_), 45.3 μM (f_5_), or 90.6 μM (f_10_) folic acid. The medium was replenished every 2 days until adipocytes had acquired intracellular lipid droplets after 12-day incubation. For the proinflammatory cytokines experiment, preadipocytes were seeded on the 24-well-plate at 1.5×10^4^ cells/well-cultured with f_0_ or f_1_ medium, and the supernatant was collected for cytokines assay after 4 days. A number of four to five independent experiments were performed for the *in vitro* folate deficiency study.

### Oil-Red O Staining

On day 16 of 3T3-L1 cell differentiation, the mature adipocytes were washed two times with PBS (pH 7.4) and then fixed with 800 μl/well of 10% paraformaldehyde for 30 min at room temperature. The cells were stained with 0.5% oil-red O staining buffer: PBS (3:2) for 40 min at room temperature. The culture plates were washed four times with PBS, followed by the microscopic images capture (Optika). Then, the plates were air-dried and added with 500 μl/well-isopropanol to dissolve lipid droplets. The lipid accumulation as the absorbance of oil-red O stained-lipid solution was determined by ELISA microplate reader (ThermoFisher) at OD_500_ nm.

### Lipid Determination

Each 100 mg liver tissue from mice at sacrifice, or mature 3T3-L1 adipocyte after 16 days of differentiation, was harvested, lysed in 5% NP-40 lysis buffer for lipid solubilization. These liver samples or 3T3-L1 cell lysates were slowly heated to 80–100°C for 5 min to ensure that lipids were extracted and dissolved. TG contents in liver tissue, or in 3T3-L1 cell lysates, and cholesterol (CHOL) contents in liver tissue were analyzed using quantification enzymatic kits (Randox, Antrim, UK), according to the manufacturer's instructions.

### Determination of Leptin Levels and Proinflammatory Cytokines

The blood samples were collected from the mice before sacrifice and then centrifuged at 12,000 rpm to obtain serum for further analysis. The medium supernatant from mature 3T3-L1 adipocytes after 16 days of differentiation was aspirated and spun down to remove debris to collect the clean supernatant. Leptin levels in sera or 3T3-L1 cell culture supernatants were assessed using the ELISA commercial kit (R&D Systems, Minneapolis, MN, USA), according to the manufacturer's protocol. Proinflammatory cytokines, monocyte chemotactic protein (MCP)-1, and interleukin (IL)-6, from 3T3-L1 preadipocyte or mature adipocyte culture supernatants, were also assessed using ELISA commercial kits (BioLegend, San Diego, CA, USA).

### RNA Extraction, Reverse Transcription, and RT-qPCR

On day 16 of 3T3-L1 cell differentiation, total RNA was extracted from adipocytes using TRIzol reagent (Invitrogen, Grand Island, NY, USA), and complementary DNA (cDNA) was synthesized using high-capacity cDNA reverse transcriptase reagent (ThermoFisher), following the manufacturer's instructions. Relative mRNA expression of the target and reference genes was determined by qPCR on a CFX Connect™ Real-Time PCR Detection System (Bio-Rad, Hercules, CA, USA) using SYBR Green supermix (Bio-Rad). Since glyceraldehyde-3-phosphate dehydrogenase (GAPDH) was stably expressed with constant Ct value in mature adipocytes cultured with or without folate, all values were normalized by a reference gene, GAPDH as internal reaction control, and further calculated using the 2^(−ΔΔCt)^ method. The sequence of primers used in qPCR was listed as follows, leptin (forward, 5′-TCT CCG AGA CCT CCT CCA TCT-3′, and reverse, 5′-TTC CAG GAC GCC ATC CAG-3′); ACC1 (forward, 5′-GAT GAA CCA TCT CCG TTG GC-3′, and reverse, 5′-CCC AAT TAT GAA TCG GGA GTG C-3′); FASN (forward, 5′-TAG CCA GCA GAG TCT ACA-3′, and reverse, 5′-TCA CAT CAG CCA CTT GAG-3′); FABP4 (forward, 5′-GGA TGG AAA GTC GAC CAC AA-3′, and reverse, 5′-TGG AAG TCA CGC CTT TCA TA-3′); C/EBPα (forward, 5′-AAC TGA GAC TCT TCA CTA ACG-3′, and reverse, 5′-ACT ACT ACA TAC ACC CTT GGA-3′); C/EBPβ (forward, 5′-CCT GCG GGG TTG TTG ATG-3′, and reverse, 5′-TCA CTT TAA TGC TCG AAA CGG AAA-3′); PPARγ (forward, 5′-GAC CTC TCC GTG ATG GAA G-3′ and reverse, 5′-GCT CTT GTG AAT GGA ATG TCT T-3′); IL-6 (forward, 5′-TCC AGT TGC CTT CTT GGG AC-3′ and reverse, 5′- GTA CTC CAG AAG ACC AGA GG-3′); HIF-1α (forward, 5′-CTA TGG AGG CCA GAA GAG GGT AT-3′ and reverse, 5′-CCC ACA TCA GGT GGC TCA TAA-3′); GAPDH (forward, 5′-AGG TCG GTG TGA ACG GAT TTG-3′ and reverse, 5′-TGT AGA CCA TGT AGT TGA GGT CA-3′). All the primer sets used in our experiment have 90–110 efficiency (%).

### Serum and Intracellular Folate Levels

Blood samples from mice fed different diets were collected at 0, 1, 2, 4.5, 5, and 12 months during the feeding period to monitor folate status. For intracellular folate contents in 3T3-L1 adipocytes, supernatants were aspirated and culture plates were washed two times with iced PBS. Then, cells were resuspended in PBS containing 0.05% ascorbic acid, followed by the sonication in an ice bath using a sonicator (Hielscher, Teltow, Germany) for 15 s and cool-down for 10 s. The cycle was repeated four times to ensure that the folate was released. Folate levels in sterile serum samples in the animal study and in 3T3-L1 cell extracts were measured using *Lactobacillus casei* (*L. casei*, ATCC 7469) microbiological assay as previously described (24). Briefly, 125 μl of diluted standard or samples and 125 μl of folic acid casei medium (Himedia) containing *L. casei* were added to the 96-well-microplate. The microplate was cultured for ~18 h at a 37°C humidified incubator and detected the absorbance at OD_600_ nm to determine the folate concentration according to the standard curve.

### Statistical Analysis

All values were expressed as mean ± SD. Statistical analysis was performed with SPSS software version 22.0 (IBM Corp., Armonk, NY, USA). All data sets were tested for normality using Shapiro–Wilk test, and the *p*-value of data sets was >0.05, indicating that data sets were normally distributed. For *in vivo* experiments, *p*-values were calculated using a one-way analysis of variance (ANOVA), followed by Duncan's *post-hoc* test. For *in vitro* experiments, significant differences were compared using an unpaired two-tailed Student's *t*-test. *P* < 0.05 were considered statistically significant.

## Results

### Serum Folate Level and Calorie Intake in Mice Fed a Folate-Deficient Diet

**Figure 2:**
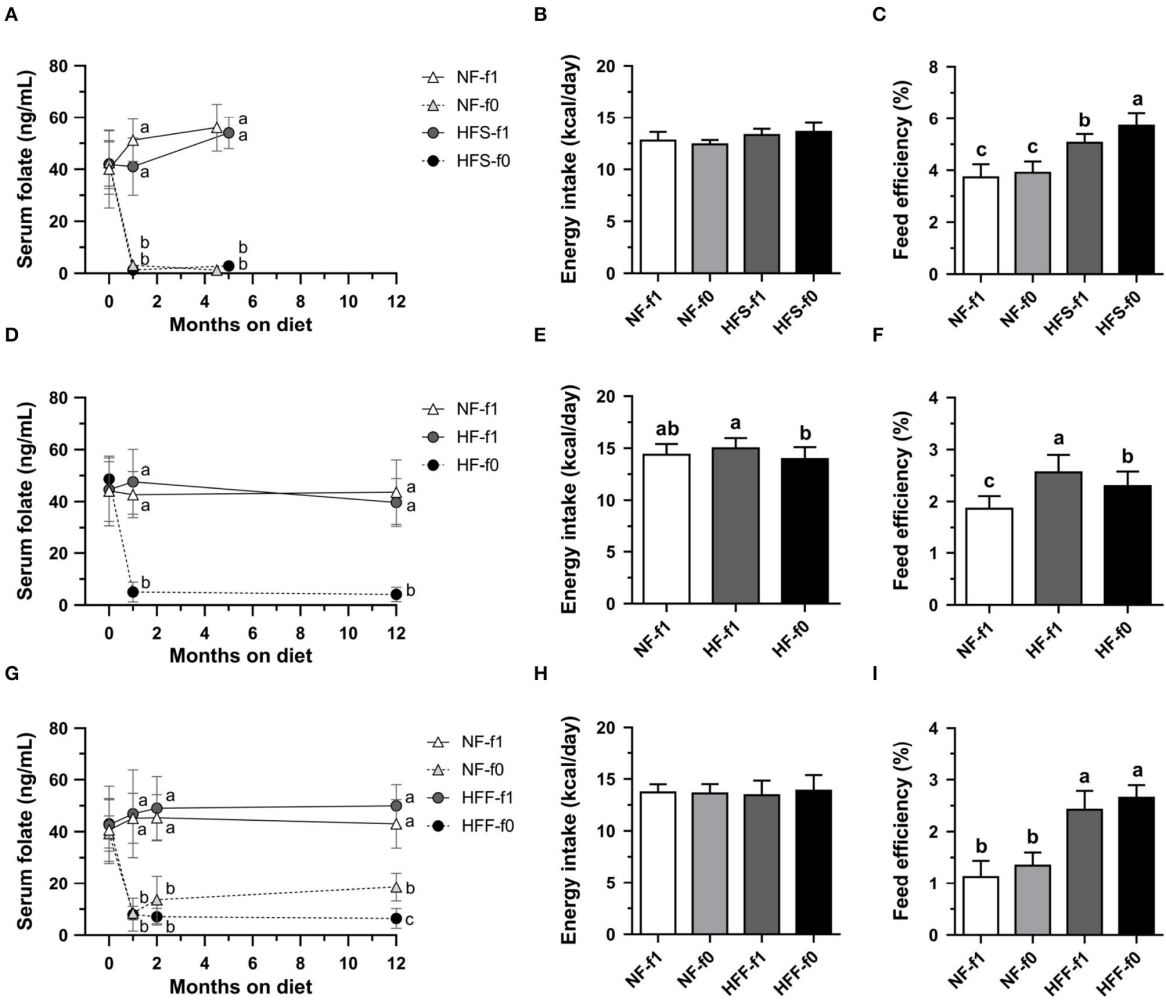
Serum folate concentration, energy intake and feed efficiency of C57BL/6 mice fed HFD (HF) containing different carbohydrate source with (f1) or without (f0) folate. **(A)** Serum folate, **(B)** energy intake, and **(C)** feed efficiency from mice fed either NFor HF containing high-sucrose (HFS) diet for 4.5–5 months. **(D–F)** from mice fed either NF or HF diet for 12 months. **(G–I)** from mice fed either NF or HF containing high-fructose (HFF) diet for 12 months. Data mean ± SD (*n* = 8–16 per group). The different letters are indicated significant differences (*p* < 0.05) among groups analyzed using one-way ANOVA and Duncan's *post-hoc* test.

To confirm the effects of folate deficiency on adiposity, sera folate levels were followed. Initial serum folate levels in 6 to 8-week-old male C57BL/6 mice fed AIN-93 NF diets with folate (NF-f1) were around 40–45 ng/ml (Figures 2A,D,G). Serum folate levels from mice fed folate-deficient diets significantly dropped to 1–3 ng/ml in the NF-f0 and HFS-f0 mice (Figure 2A). The daily calorie intakes were similar among the NF and HFS groups with or without folate, but the HFS-f0 diet had higher feed efficiency than the HFS-f1 diet during 5-month feeding (Figures 2B,C).

Serum folate levels from mice fed a folate-deficient diet significantly dropped to around 5 ng/ml in the HF-f0 mice (Figure 2D). Long-term folate deficiency in HFD (HF-f0) decreased calorie intake and feed efficiency when comparing the HF-f1 diet after 12-month feeding (Figures 2E,F).

When mice were fed a high-fat high-fructose diet for 12 months, serum folate was around 14–18 ng/ml in the NF-f0 group after the addition of 0.2 mg folate to the NF-f0 diet, and 5 ng/ml in the HFF-f0 mice (Figure 2G). There was no difference in calorie intake among the NF-f1, NF-f0, HFF-f1, and HFF-f0 groups (Figure 2H). Feed efficiencies were also not significantly affected by folate deficiency (Figure 2I).

### Folate Deficiency Increased Adipose Tissue and Serum Leptin Level in Mice

**Figure 3:**
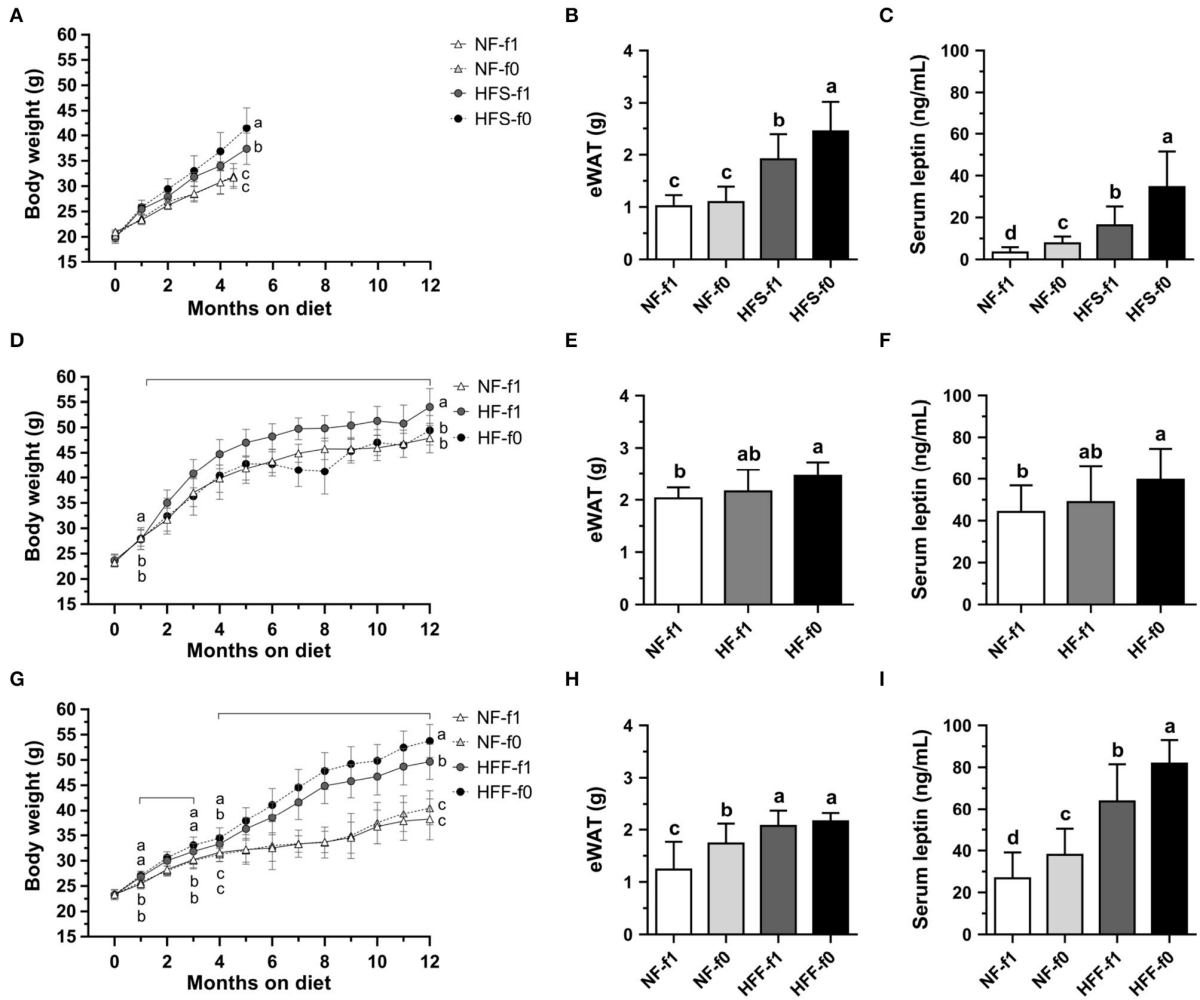
Folate deficiency increased adipose tissue mass and sera leptin levels of mice fed either NF, high-fat high-sucrose (HFS), high-fat (HF), or high-fat high-fructose (HFF) diet for 4.5–5 or 12 months. **(A)** Body weight, **(B)** epididymal white adipose tissue (eWAT) mass, and **(C)** serum leptin from NF or HFS diet-fed mice. **(D–F)** from NF or HF diet-fed mice. **(G–I)** from NF or HFF diet-fed mice. Data are mean ± SD (*n* = 8–16 per group). The different letters indicate significant differences (*p* < 0.05) analyzed using one-way ANOVA and Duncan's *post-hoc* test.

To investigate whether folate deficiency affects adipose tissue formation, we fed mice either NF or high-fat high-sucrose (HFS) diet with (NF-f1, HFS-f1) or without folate (NF-f0, HFS-f0). Folate deficiency did not affect body weight and epididymal white adipose tissue (eWAT) in NF diet-fed mice for 4.5 months (Figures 3A,B). However, sera leptin levels were significantly higher in the NF-f0 group mice compared to those of the NF-f1 group mice (Figure 3C), indicating that folate deficiency increased serum leptin though no difference in body weight and eWAT was observed in the short-term, 4.5-month feeding with NF diet.

When mice were fed with an HFS diet, which mimicked the high-sugar intake of modern society, folate deficiency enhanced diet-induced obesity in 5-month feeding. The HFS-f0 mice had significantly higher body weight, eWAT mass, and serum leptin level compared to the HFS-f1 mice (Figures 3A–C). Our results indicated that short-term dietary folate deficiency might increase adiposity and circulating leptin level of mice, especially combined with a high-fat high-sucrose diet.

To further examine the effects of long-term folate deficiency in HFD on the development of obesity, mice were fed either NF-f1, HF-f1, or HF-f0 diet for 12 months. Although the HF-f0 mice had lower body weight than the HF-f1 mice (Figure 3D), HF-f0 mice trended to have higher eWAT mass and serum leptin levels than the HF-f1 mice (Figures 3E,F) and significantly higher than the NF-f1 mice. The results suggested that folate deficiency additionally increased adiposity induced by the HF diet.

When corn starch in the HF diet was replaced with fructose, mice fed a high-fat high-fructose (HFF) diet with folate deficiency significantly increased body weight and serum leptin (Figures 3G,I). In order not to decrease the appetite of NF-fed mice due to folate deficiency, the NF-f0 diet was added one-tenth of f1 folate content (0.2 mg folic acid/kg diet). As shown in Figures 3H,I, the NF-f0 mice had significantly higher eWAT mass and serum leptin than the NF-f1 mice. Sera leptin levels were also significantly higher in HFF-f0 mice compared to the HFF-f1 mice although no difference in eWAT mass was observed in the HFF groups. Our results demonstrated that dietary folate insufficiency might enhance the development of adiposity.

### Folate-Deficient HF or HFF Diet Increased Adipocyte Size and Weight in Mice

**Figure 4:**
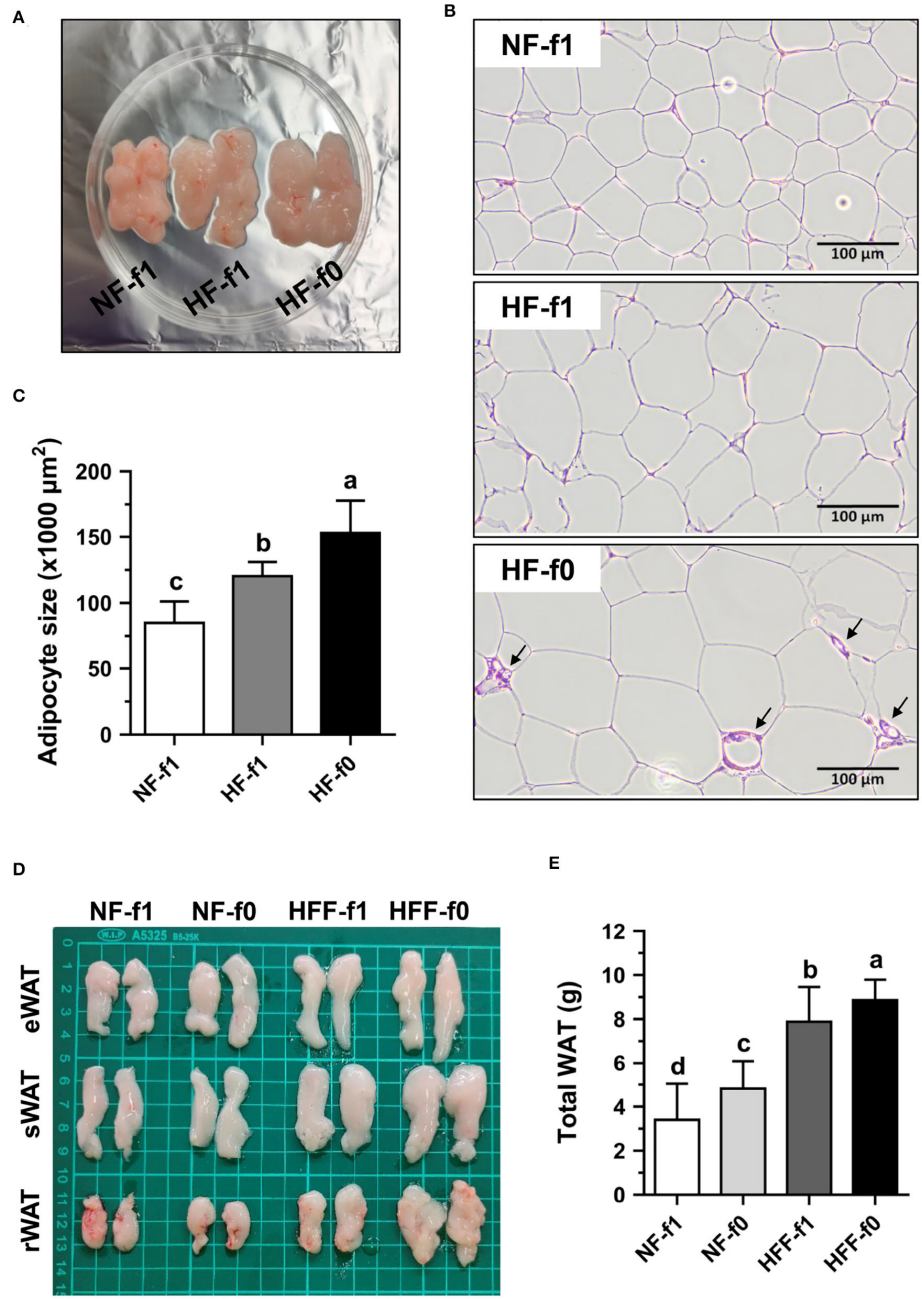
Folate deficiency increased adipocyte size of mice fed with high-fat (HF) and fat pad of mice fed either NF or high-fat high-fructose (HFF) diet for 12 months. The representative images of **(A)** epididymal WAT (eWAT) fat pad, **(B)** hematoxylin and eosin (H&E)-stained eWAT, the arrow indicates fibrotic lesion, and **(C)** the quantification of adipocyte size in eWAT from NF or HF diet-fed mice. The representative images of **(D)** eWAT, subcutaneous WAT (sWAT) and perirenal WAT (rWAT) fat pads, and **(E)** total weight of WAT from NF or HFF diet-fed mice. Data are mean ± SD (*n* = 10–16 per group). Bars with different letters indicate significant differences (*p* < 0.05) analyzed using one-way ANOVA and Duncan's *post-hoc* test.

Since folate is necessary for cell division and the HF-f0 mice showed a tendency of higher eWAT than the HF-f1 mice, adipocytes size in eWAT was further investigated. As shown in Figure 4A, the eWAT fat pad of HF mice showed larger than that of the NF mice. H&E staining of eWAT sections showed the larger adipocyte size in both the HF groups mice and markedly fibrotic lesions in eWAT from the HF-f0 mice (Figure 4B). The adipocyte size of eWAT from HF-f0 mice was significantly higher than those of the HF-f1 mice after quantification using ImageJ software (Figure 4C).

Since eWAT mass was not different between the HFF-f0 mice and the HFF-f1 mice, four types of fat tissues, eWAT, sWAT, rWAT, and mWAT, were collected and weighed. The total weight of WAT as the sum of the weights of these WATs demonstrated that folate deficiency for 12 months significantly increased fat mass in both the NF and HFF groups mice (Figures 4D,E). These results confirmed that dietary folate deficiency increased adipocyte size and adipose tissue mass in mice.

**Figure 5:**
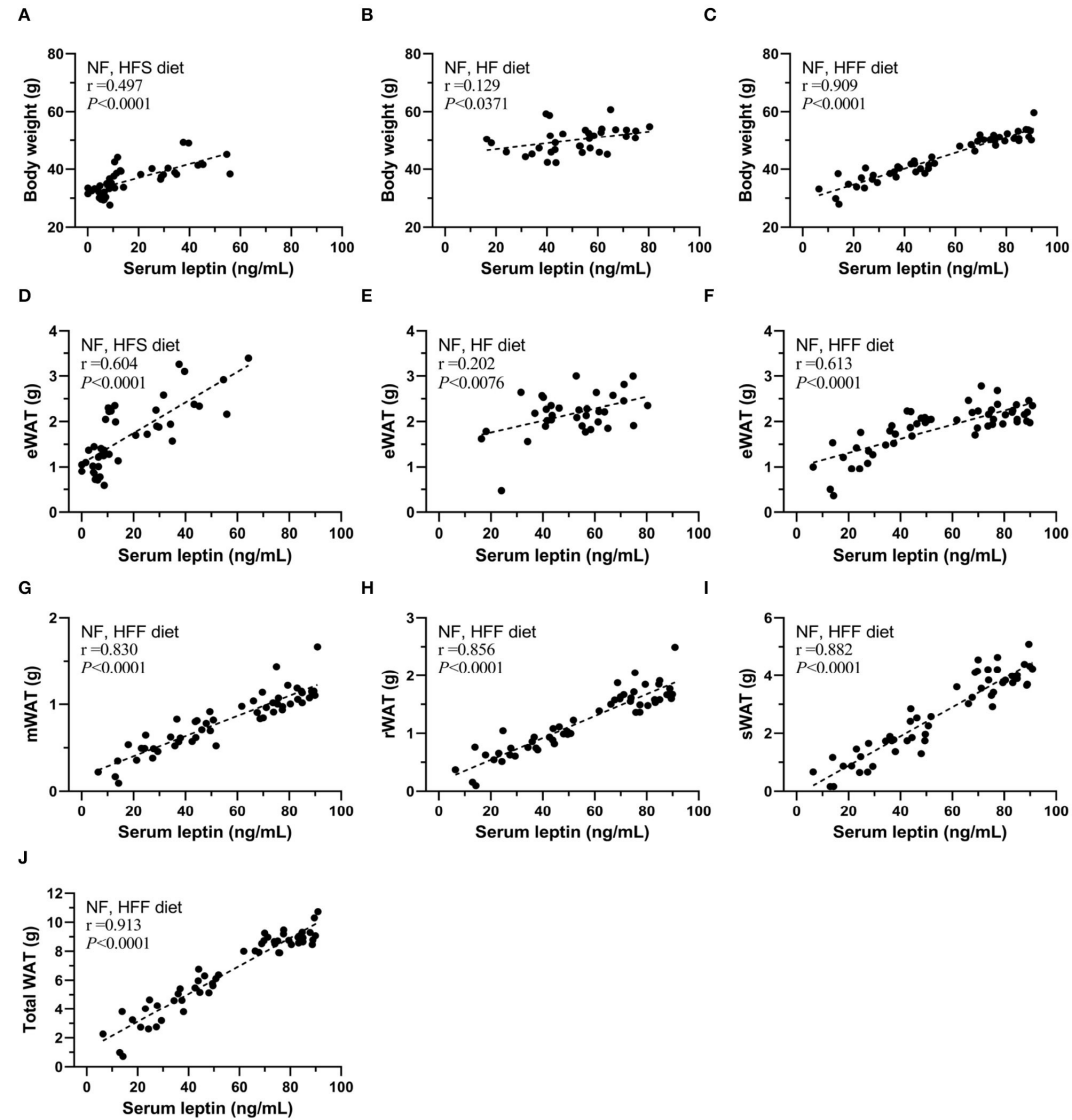
Serum leptin levels correlated with body weight and WAT mass of mice of each group. The correlation between serum leptin and body weight of **(A)** NF and high-fat high-sucrose (HFS) groups, **(B)** NF and HF groups, and **(C)** NF and high-fat high-fructose (HFF) groups. The correlation between serum leptin and epididymal WAT (eWAT) of **(D)** NF and HFS groups, **(E)** NF and HF groups, and **(F)** NF and HFF groups. The correlation between serum leptin and **(G)** mesenteric WAT (mWAT), **(H)** perirenal WAT (rWAT), **(I)** subcutaneous (sWAT), **(J)** total WAT of NF and HFF groups. Data are mean ± SD (*n* = 8–16 per group). The correlation coefficient was analyzed by the Pearson's correlation and represented as r value (*p* < 0.05).

In addition, sera leptin levels were positively correlated with body weight, eWAT in these HFS, HF, and HFF feeding experiments (Figures 5A–F), especially the strong correlation in the HFF experiment for 12-month feeding. Therefore, we further analyzed the correlation of serum leptin with different parts of WAT of the HFF mice (Figures 5F–J). Sera leptin levels were strongly correlated with all WAT isolated from different locations. The correlations with serum level of leptin were presented as an ascending order from eWAT (r = 0.613), mWAT (r = 0.830), rWAT (r = 0.856), and sWAT (r = 0.882), and total WAT mass and had the strongest correlation with serum leptin (r = 0.913).

### Folate Deficiency Promoted Hepatic Lipid Accumulation and Progression of Steatosis in Mice

**Figure 6:**
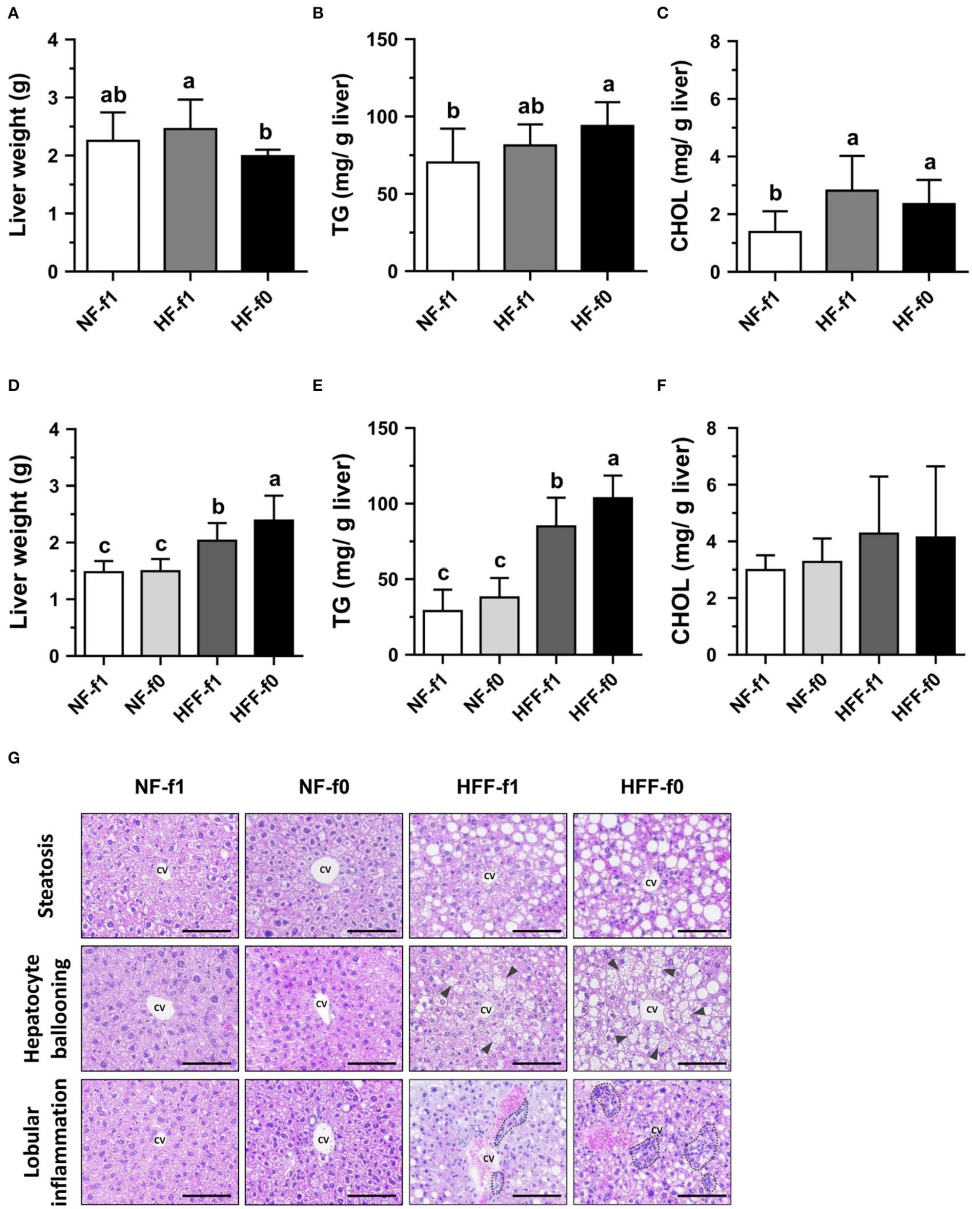
Folate deficiency increased hepatic lipid accumulation of mice fed either high-fat (HF) or high-fat high-fructose (HFF) diet for 12 months. Liver **(A)** weight, **(B)** TG content, and **(C)** cholesterol (CHOL) content from NF or HF diet-fed mice, and **(D–F)** from NF or HFF diet-fed mice, respectively. **(G)** The representative images of H&E-stained liver (scale bar: 100 μm) from HFF diet-fed mice, the triangle indicates hepatocellular ballooning, and the dotted area indicates infiltration of mononuclear leukocytes. Data are mean ± SD (*n* = 10–16 per group). Bars with different letters indicate the significant difference (*p* < 0.05) analyzed using one-way ANOVA and Duncan's *post-hoc* test. CV, central vein.

Since the liver is an essential organ for all metabolic processes and regulation of the levels of lipid, glucose, and energy metabolism, hepatic lipid accumulation and morphology were investigated. Despite that the HF-f0 mice had the lowest liver weight among the three groups (Figure 6A), the HF-f0 mice had the highest hepatic TG contents (Figure 6B). The hepatic cholesterol (CHOL) increased by the HF diets but did not statistically differ between the HF-f1 and HF-f0 groups (Figure 6C).

When mice were fed HFF diets, folate deficiency on hepatic lipid accumulation became significant. The HFF diets significantly increased liver weight and hepatic TG contents in mice than the NF diets (Figures 6D,E). No difference was observed in liver weight, hepatic TG, and cholesterol contents in the NF-fed mice. However, HFF-f0 mice had significantly higher liver weight and hepatic TG than the HFF-f1 mice. Hepatic cholesterol contents did not reach a significant difference among the groups (Figure 6F).

To further investigate the effects of folate deficiency on the liver, liver tissue sections were stained with H&E for the observation of morphological changes. The HFF diet-fed mice had more steatosis, hepatocyte ballooning, and lobular inflammation compared to the NF diet-fed mice (Figure 6G). Furthermore, the folate-deficient diets had more severe morphological changes (HFF-f0 vs. HFF-f1). Our results indicated that dietary folate deficiency increased hepatic lipid accumulation and exacerbated pathological changes in mice.

### Higher Lipid and Leptin Production by Folate Deficiency in 3T3-L1 Adipocytes

**Figure 7:**
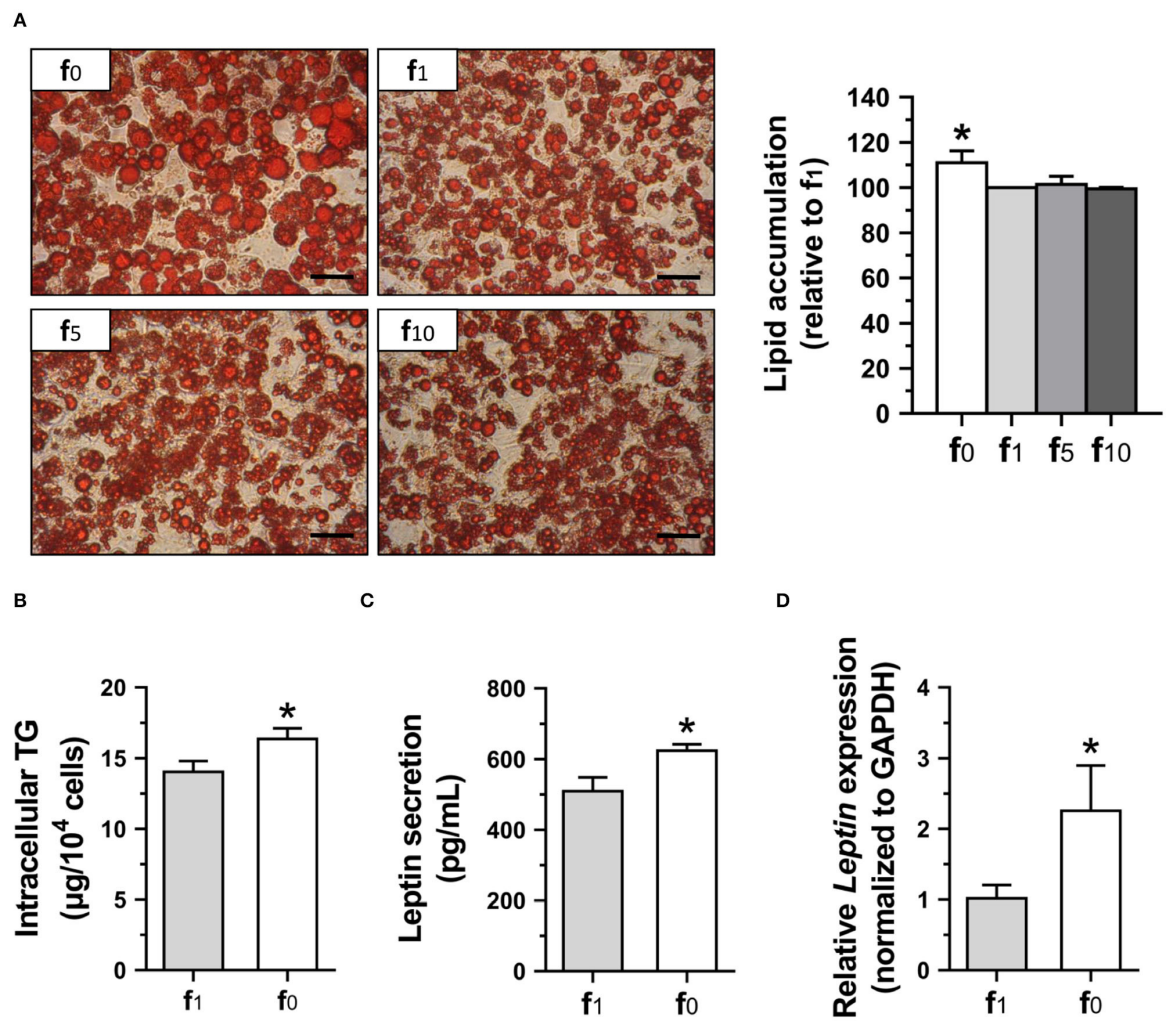
Folate deficiency enhanced lipid accumulation and leptin secretion of 3T3-L1 adipocytes. **(A)** The representative images of oil-red O-stained adipocytes (scale bar: 100 μm) and lipid accumulation, **(B)** intracellular TG content, **(C)** leptin secretion, and **(D)** leptin gene expression determined using qPCR assay. Data mean ± SD (*n* = 4). *p < 0.01 vs. f_1_ group by Student's *t*-test.

To confirm the effects of folate deficiency on lipid accumulation and leptin secretion of mature adipocytes, 3T3-L1 cells were cultured for 12 days with either folate-deficient (f_0_), or different folate concentrations (f_1_, f_5_, f_10_) in the differentiation medium. The mature 3T3-L1 adipocytes stained with oil-red O for lipid droplets are shown in Figure 7A. The lipid droplets were larger and lipid accumulation was higher in folate-deficient 3T3-L1 adipocytes. There was no significant difference among media with various folate concentrations (f_1_, f_5_, f_10_). Therefore, the intracellular TG contents of these 3T3-L1 adipocytes cultured in f_0_ or f_1_ were determined and confirmed significantly higher intracellular TG in folate-deficient 3T3-L1 adipocytes (Figure 7B).

Since leptin level has been shown to be correlated with fat mass and adipocyte size, leptin contents in the medium supernatant from mature 3T3-L1 adipocyte cultures were also analyzed. The leptin secretion in folate-deficient adipocytes was significantly higher (Figure 7C). To further confirm the effects of folate deficiency on leptin, leptin (*Leptin*) mRNA expression in 3T3-L1 adipocytes was detected and also shown to have significantly higher gene expression of leptin when cultured with an f_0_ differentiation medium (Figure 7D).

### Higher Level of Proinflammatory Cytokine and Lipogenic Gene Expression in Folate-Deficient 3T3-L1 Adipocytes

**Figure 8:**
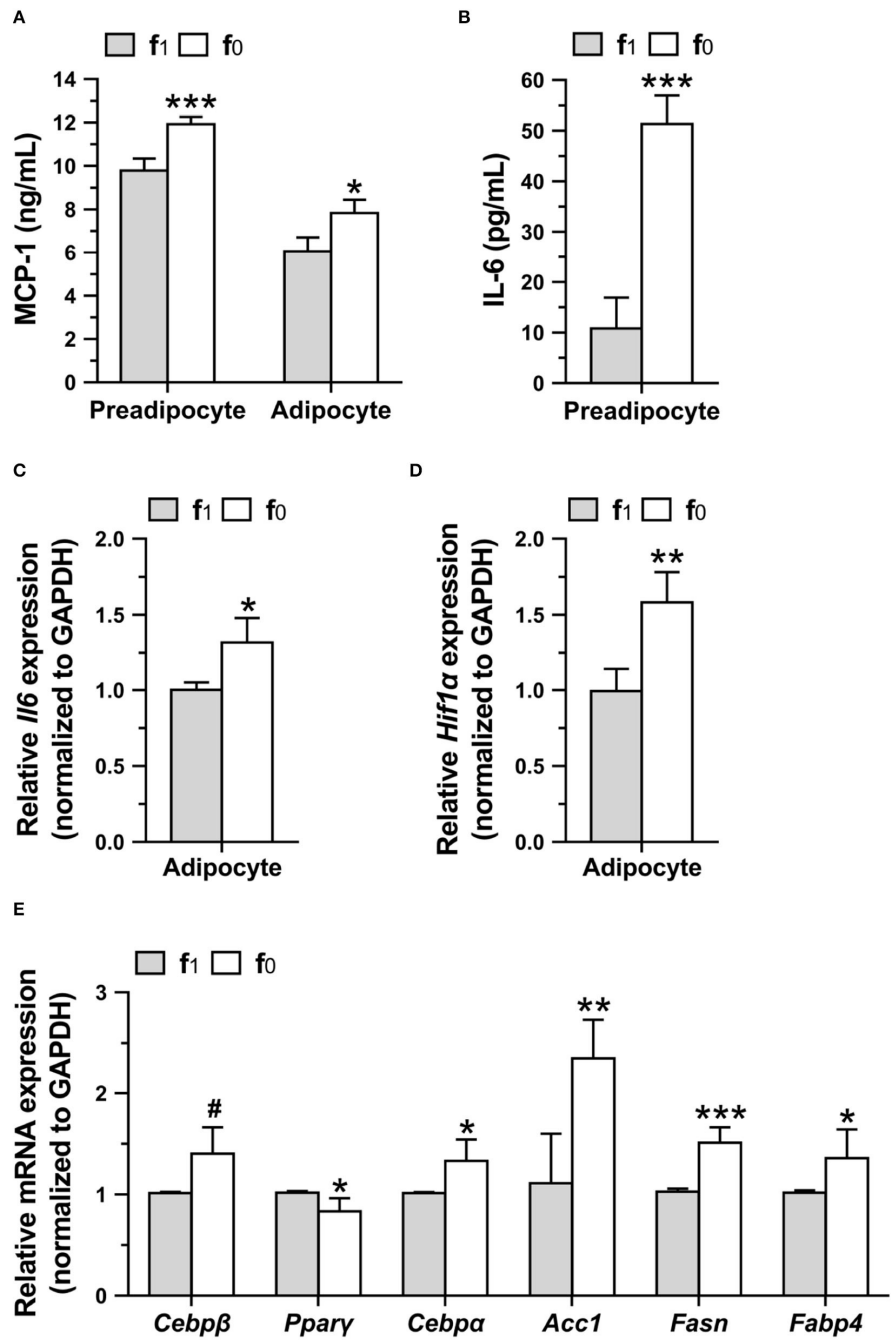
Folate deficiency increased proinflammatory cytokine productions and lipogenic gene expressions of 3T3-L1 cells. Preadipocytes were cultured in medium with (f_1_) or without (f_0_) folate for 4 days, and adipocytes were differentiated for 16 days. **(A)** MCP-1 and **(B)** IL-6 secretions by preadipocytes or adipocytes were analyzed using ELISA. The relative expressions of **(C)** *Il6* and **(D)** *Hif1*α, and **(E)** lipogenic genes in adipocytes were performed by qPCR assay. Data are mean ± SD (*n* = 4). ^#^0.05 < *p* < 0.1, *p < 0.05, **p < 0.01, and ***p < 0.001 vs. f_1_ group by Student's *t*-test.

To investigate whether higher leptin levels in folate-deficient 3T3-L1 cells might affect proinflammatory cytokine secretion, MCP-1 and IL-6 levels in preadipocyte and mature adipocytes differentiated in a medium with (f_1_) or without folate (f_0_) were measured. Folate-deficient 3T3-L1 preadipocytes and adipocytes had significantly higher MCP-1 secretion (Figure 8A). Higher IL-6 secretion of preadipocytes and expression of *Il6* and *Hif1*α genes of adipocytes were also detected when cultured with an f_0_ medium (Figures 8B–D).

To elucidate higher lipid accumulation in folate-deficient adipocytes, the expression of lipogenic genes in mature differentiated 3T3-L1 adipocytes was analyzed. Real-time qPCR assays confirmed that folate deficiency significantly increased the expression of lipogenic-related genes (Figure 8E). Tendentiously higher expressions of *Cebp*β that are expressed in the early phase of differentiation to regulate lipid accumulation and lower expression of *Ppar*γ that are expressed in an intermediate phase were detected in folate-deficient 3T3-L1 adipocytes. From an intermediate to late phase of differentiation, folate deficiency significantly enhanced the expression of *Cebp*α, a transcription factor that binds leptin promotor, and also increased the expression of acetyl-CoA carboxylase 1 (*Acc1*) and FASN, the key enzymes in the *de novo* fatty acid biosynthetic pathway. The expression of fatty acid-binding protein-4 (*Fabp4*), an intracellular lipid transporter, was also enhanced by folate deficiency. These results indicated that folate deficiency might enhance the lipid accumulation in adipocytes by promoting lipogenic gene expressions.

## Discussion

Our results revealed that short-term or long-term dietary folate deficiencies significantly increased lipid accumulation and leptin secretion of adipocytes. Folate-deficient C57BL/6 mice were confirmed by the significantly lower serum folate level after 1-month feeding of a folate-deficient diet. Though energy intake of mice was not significantly different between the NF and HFS groups mice, feed efficiency and body weight were significantly higher in folate-deficient mice (HFS-f0) than that of the folate-sufficient mice (HFS-f1) after 5-month feeding. A study also showed significantly higher body weight and eWAT in Institute of Cancer Research (ICR) mice fed with a folate-deficient NF diet for 6 months (25), with serum folate levels of 4.02 ng/ml. These results suggested that folate deficiency might increase fat accumulation. However, for a longer feeding, a study reported that CBA/Ca mice fed NF folate-deficient (0.1–0.2 mg/kg diet) diet for 8 months, with serum folate levels around 5 ng/ml, had lower body weight gain (26). Our study also showed that C57BL/6 mice fed the HF-f0 diet for 12 months had significantly lower energy intake, feed efficiency, and thus lower body weight compared to the HF-f1 mice. This might be explained by the loss of appetite and impaired intestinal mucosal cells by folate deficiency. Malnutrition affected the survival of the NF-f0 mice in this long-term HF experiment. Therefore, to keep NF-f0 mice surviving in the following HFF experiment, one-tenth of f1 folate (0.2 mg/kg diet) was added to the NF-f0 diet after 1 month of feeding when the feed intake and body weight of the NF-f0 mice dropped dramatically. The NF-f0 mice restored feed intake and survived with serum folate levels of around 14 ng/ml.

Interestingly, folate deficiency decreased feed intake and thus body weight of the HF mice, but still slightly increased eWAT and cell size of adipose tissue in the HF-f0 mice compared with those of HF-f1 mice. When mice fed HFDs with either high-sucrose or high-fructose (HFS-f0, HFF-f0), there is no difference in feed intake between the f1 and the f0 mice, which might be due to the sweet taste preference of C57BL/6 mice (27). However, the f0 mice groups still had higher body weights than the f1 mice groups. In addition, when energy intake was similar, the enhancing effect of folate deficiency on fat accumulation is also shown in the NF diet after long-term feeding. Our results indicated that folate deficiency enhances adipocyte expansion due to hypertrophic adipocytes. Higher leptin secretion and hypertrophy-induced local adipose tissue hypoxia stress increased local inflammation (4, 5). Higher MCP-1 and IL-6 levels in folate-deficient 3T3-L1 cells suggested that adipocytes hypertrophy and inflammation might contribute to promoted diet-induced adiposity by folate deficiency.

*In vitro* study also confirmed that 3T3-L1 adipocytes cultured without folate had higher lipid accumulation and TG contents. Our result is consistent with the report that methotrexate, an anti-folate drug, enhanced 3T3-L1 adipocytes hypertrophy (28). It might be explained that the expressions of lipogenic genes, such as *Cebp*β, *Cebp*α, *Acc1, Fasn*, and *Fabp4*, were promoted by folate deficiency. In accordance with lipogenic genes, leptin secretion and gene expression were significantly higher in folate-deficient 3T3-L1 adipocytes. There are few studies investigating the effects of micronutrient deficiencies on leptin production. Vitamin A (retinoic acid)-deficient mice had higher leptin mRNA expression (29), but zinc deficiency reduced leptin gene expression and secretion in rat adipocytes (30), suggesting that the effect of micronutrients on leptin and other adipokines still needs to be explored. In this study, we demonstrated that folate deficiency increased serum level of leptin in mice fed with folate-deficient diets, and in folate-deficient 3T3-L1 mature adipocytes, indicating that folate deficiency or insufficiency might contribute to the increase in obesity prevalence and further chronic inflammatory disease. So far, almost 80 countries have introduced mandatory fortification of bread-making flour with folic acid, but more than 100 countries, such as European Union countries, are still not implementing this policy (31). Since many factors in addition to folate deficiency might affect the prevalence of overweight and obesity, there are no epidemiological data showing the effects of folate-fortified flour on the prevalence of overweight and obesity. However, it is noteworthy that adequate folate status is also important for obesity prevention, especially in those countries without folic acid fortification.

There was no significant difference in energy intake, feed efficiency, body weight, and eWAT of the NF mice in short-term feeding. However, serum leptin concentration was significantly higher in the NF-f0 mice than those of the NF-f1 mice, despite the feeding period, suggesting that serum leptin levels might be sensitive indicators of fat mass accumulation in the body. A significant correlation between serum leptin and body weight and WAT was also observed in our study. Sera leptin levels were also higher in the folate-deficient mice fed with HFDs, especially in the high-fructose diet. The stronger correlation between serum leptin and body weight in the HFF experiment indicated that fructose also plays an important role in adipogenesis.

To investigate whether folate deficiency exerts promoting effect on lipogenesis, we replaced corn starch in the HF diet with fructose in the HFF experiment. Fructose is mainly metabolized by the liver which is also a metabolically active organ for lipid metabolism (32). Since fructose is dominantly metabolized to fructose-1-phosphate by fructokinase that is not regulated by its byproducts, fructose bypasses the regulated steps of glycolysis and thus promotes *de novo* lipogenesis (33). One study demonstrated that mice on HFD supplemented with fructose for 10 weeks had higher liver weight and hepatic mRNA expression of fructokinase and FASN than glucose-supplemented mice, suggesting the promoting effect of fructose on hepatic lipogenesis (23). They showed no difference in liver TG between these two groups, but higher body weight and visceral fat/total fat in the fructose-supplemented group. This suggested that excessive hepatic lipids might be exported through lipoprotein particles toward the peripheral organs (34). Our results also showed similar liver TG between the HF-f1 and the HFF-f1 mice after 12-month feeding. However, folate deficiency further increased liver TG in the HF-f0 mice despite the lower liver weight, as compared to the HF-f1 mice, and significantly increased both liver TG and liver weight in the HFF diet. In addition to enhancing fatty acid synthesis, folate deficiency also decreases the methylation and thus the formation of phosphatidylcholine, leading to less very low-density lipoprotein produced for hepatic lipid efflux (25). Therefore, an increasing number of evidence suggested the association between folate status with non-alcoholic fatty liver disease in the recent review articles (35, 36). In our study, folate-deficient mice also had more steatosis, hepatocytes ballooning, and lobular inflammation, suggesting that chronic lipid accumulation enhanced by folate deficiency might result in pathological changes in the liver. Serum folate levels achieved are extraordinarily low which might limit public health significance. However, the results here could still provide useful information for future studies on human subjects with folate deficiency and obesity.

In conclusion, our results demonstrated that dietary folate deficiency increased lipid accumulation and leptin secretion of adipocytes both *in vitro* and *in vivo*. Therefore, folate deficiency might be one of the most important risk factors for obesity development. It should be more emphasized that adequate micronutrient intake is extremely important to maintain body health and physical homeostasis.

## Data Availability Statement

The original contributions presented in the study are included in the article/Supplementary Material, further inquiries can be directed to the corresponding author.

## Ethics Statement

The animal study was reviewed and approved by Institutional Animal Care and Use Committee (IACUC) of the National Taiwan University (approval number: NTU105-EL-17 and NTU107-EL-235).

## Author Contributions

B-FL and C-WC planned, designed, conducted the research, and were the major contributors in writing the manuscript. C-WC and P-HC carried out the animal experiments and data analyses under the supervision of B-FL. C-WC also performed the cell culture experiments. All authors approved the final manuscript for publication.

## Funding

B-FL was funded by the Ministry of Science and Technology Science Council of the Republic of China, grants number MOST105-2320-B-002-028-MY3 and MOST108-2320-B-002-069-MY3.

## Conflict of Interest

The authors declare that the research was conducted in the absence of any commercial or financial relationships that could be construed as a potential conflict of interest.

## Publisher's Note

All claims expressed in this article are solely those of the authors and do not necessarily represent those of their affiliated organizations, or those of the publisher, the editors and the reviewers. Any product that may be evaluated in this article, or claim that may be made by its manufacturer, is not guaranteed or endorsed by the publisher.
