# Supplementary material for: Folate Deficiency Increased Lipid Accumulation and Leptin Production of Adipocytes
Source: Front Nutr. 2022 Apr 25;9:852451. doi: 10.3389/fnut.2022.852451 (PMC9083361; doi:10.3389/fnut.2022.852451)
Supplement: Supplementary file 1 [file Table_1.DOCX]

# Supplementary Material

**Supplementary Table 1.** AIN-93G Purified Diet Composition^1^

| Ingredients  (g/kg diet) | Experiment 1, 2, 3 | | Experiment 1 | | Experiment 2 | | Experiment 3 | |
| --- | --- | --- | --- | --- | --- | --- | --- | --- |
|  | **NF-f1** | **NF-f0**^2,3^ | **HFS-f1** | **HFS-f0**^2^ | **HF-f1** | **HF-f0** | **HFF-f1** | **HFF-f0** |
| Folic acid (mg) | 2 | 0.2 | 2 |  | 2 |  | 2 |  |
| Butter |  |  | 290 | 290 |  |  |  |  |
| Lard |  |  |  |  | 226 | 226 | 226 | 226 |
| Soybean oil | 70 | 70 | 10 | 10 | 10 | 10 | 10 | 10 |
| Corn starch | 529.5 | 529.5 |  |  | 316 | 316 |  |  |
| Sucrose | 100 | 100 | 321 | 321 | 100 | 100 | 100 | 100 |
| Fructose |  |  |  |  |  |  | 316 | 316 |
| Casein | 200 | 200 | 254 | 254 | 230 | 230 | 230 | 230 |
| L-Cysteine | 3 | 3 | 4 | 4 | 4 | 4 | 4 | 4 |
| Cellulose | 50 | 50 | 61 | 61 | 57 | 57 | 57 | 57 |
| Mineral mix | 35 | 35 | 44.5 | 44.5 | 42 | 42 | 42 | 42 |
| Vitamin mix  (w/o folic acid) | 10 | 10 | 12.5 | 12.5 | 12 | 12 | 12 | 12 |
| Choline Bitartrate | 2.5 | 2.5 | 3 | 3 | 3 | 3 | 3 | 3 |
| Total energy, kcal | 3960 | 3960 | 5016 | 5016 | 4724 | 4724 | 4724 | 4724 |
| Fat (% kcal) | 15.9 | 15.9 | 53.8 | 53.8 | 45.0 | 45.0 | 45.0 | 45.0 |
| CHO (% kcal) | 63.6 | 63.6 | 25.6 | 25.6 | 35.2 | 35.2 | 35.2 | 35.2 |
| Protein (% kcal) | 20.5 | 20.5 | 20.6 | 20.6 | 19.8 | 19.8 | 19.8 | 19.8 |

1. Reeves, P. G., Nielsen, F. H., & Fahey, G. C. (1993). AIN-93 Purified Diets for Laboratory Rodents: Final Report of the American Institute of Nutrition Ad Hoc Writing Committee on the Reformulation of the AIN-76A Rodent Diet. *Journal of Nutrition*, 123(11), 1939-1951
2. Antibiotics (succinylsulfathiazole, 10 g/kg diet) were added in experiment 1.
3. Folic acid (0.2 mg/kg diet) was added in NF-f0 diet after one month feeding only in experiment 3.
